# Patient and healthcare professional perceptions of colostomy‐related problems and their impact on quality of life following rectal cancer surgery

**DOI:** 10.1002/bjs5.69

**Published:** 2018-05-07

**Authors:** H. Elfeki, A. Thyø, D. Nepogodiev, T. D. Pinkney, M. White, S. Laurberg, P. Christensen, the Colostomy Impact Score study group, A. D'Hoore, E. Espin, K. Buzatti, M. Wong, N. Smart, N. Figueiredo, P. Nilsson, R. Madoff, S. Wexner, T. Cecil, T. Øresland, W. Omar, W. Borstlap

**Affiliations:** ^1^ Colorectal Surgical Unit, Department of Surgery Aarhus University Hospital Aarhus Denmark; ^2^ Colorectal Surgical Unit, Department of Surgery Mansoura University Hospital Mansoura Egypt; ^3^ Academic Department of Surgery, University of Birmingham Birmingham UK; ^4^ Colorectal Surgery Department, Queen Elizabeth Hospital University Hospitals Birmingham NHS Foundation Trust Birmingham UK; ^5^ Belgium; ^6^ Spain; ^7^ Brazil; ^8^ Singapore; ^9^ UK; ^10^ Portugal; ^11^ Sweden; ^12^ USA; ^13^ USA; ^14^ UK; ^15^ Norway; ^16^ Egypt; ^17^ The Netherlands

## Abstract

**Background:**

The perception of colostomy‐related problems and their impact on health‐related quality of life (QoL) may differ between patients and healthcare professionals. The aim of this study was to investigate this using the Colostomy Impact Score (CIS) tool.

**Methods:**

Healthcare professionals including consultant colorectal surgeons, stoma nurses, ward nurses, trainees and medical students were recruited. An online survey was designed. From the 17 items used to develop the CIS, participants chose the seven factors they thought to confer the strongest negative impact on the QoL of patients with a colostomy. They were then asked to rank the 12 responses made by patients to the final seven factors contained in the CIS. Results were compared with the original patient rankings at the time of development of the CIS.

**Results:**

A total of 156 healthcare professionals (50·4 per cent of the pooled professionals) from 17 countries completed the survey. Of the original seven items in the CIS, six were above the threshold for random selection. Ranking the responses, a poor match between participants and the original score was detected for 49·7 per cent of the professionals. The most under‐rated item originally present in the CIS was stool consistency, reported by 47 of the 156 professionals (30·1 per cent), whereas frequency of changing the stoma bag was the item not included in the CIS that was chosen most often by professionals (124, 79·5 per cent). Significant differences were not observed between different groups of professionals.

**Conclusion:**

The perspective of colostomy‐related problems differs between patients with a colostomy and healthcare professionals.

## Introduction

Surgery for colorectal cancer results in a planned permanent stoma in 10–30 per cent of patients^1^, ^2^, ^3^. The overall complication rate after stoma surgery varies from 21 to 70 per cent; complications include flux, retraction, stenosis and parastomal herniation^4^. Patients with a stoma also deal with daily stoma‐related practical management issues such as stool leakage and odour. Having a stoma can change a patient's perception of body image. Several studies^5^, ^6^, ^7^ have described a negative impact on quality of life (QoL) among patients with a stoma following surgery for colorectal cancer.

Healthcare professionals dealing with these patients should have knowledge and understanding of stoma‐related problems and their potential impact on patients' QoL. They should have a good appreciation of patients' perceptions of these problems. This should improve preoperative counselling and post‐treatment management.

The Colostomy Impact Score (CIS) (*Appendix S1*, supporting information) has recently been devised for patients left with a permanent stoma after rectal cancer surgery^8^. According to the responses obtained from each of 610 Danish patients with a colostomy who were included in a nationwide cohort study, items that contributed significantly to reduced QoL were selected for the development and validation of the CIS. The CIS is weighted to evaluate the aspects of colostomy‐related problems that have a negative impact on QoL from the patients' point of view. It was developed based on the results of 17 relevant items in the Basic Stoma Questionnaire^2^. Logistic regression analyses identified and selected items for the CIS, and multivariable analysis established the score values allocated to each item.

The aim of the present study was to detect differences in the perception of the relative impact of colostomy‐related problems on QoL between patients and healthcare professionals using the CIS. The study was conducted before publication of the CIS.

## Methods

Healthcare professionals and medical students were asked to rate their perception of stoma‐related problems, and responses were compared with data obtained from patients when the CIS was created.

Fourteen centres around the world involved in collaborative research with the Department of Surgery at Aarhus University Hospital were identified. An invitational e‐mail was sent to the leading colorectal consultant in each centre, explaining the study and asking for nomination and e‐mail addresses of another consultant, two dedicated trainees, two stoma nurses and two ward nurses. Medical students were recruited from the medical students' network (EuroSurg Students). Additionally, stoma nurses were approached via the Association of Stoma Care Nurses (ASCN UK).

The survey was set up using the online data capture system Research Electronic Data Capture (REDCap)^9^. This allowed the administration of links and direct entry of data into a combined database. Selected healthcare personnel received an e‐mail with an invitation letter, which also explained the study. After they had agreed to participate, they were given a link to the survey where they completed a few background questions about their profession, years of experience, country of practice and whether they had previously seen the finalized CIS.

In step 1, participants were offered a list of 17 questions (identical to the 17 items presented to patients for the development of the CIS, in the same order) (*Appendix S2*, supporting information) and asked to choose the seven items that in their opinion would have the greatest negative impact on QoL for patients with a colostomy. Their choices were compared with those derived from patients for the CIS.

In step 2, participants were given another task. The seven factors in the CIS (patient choices) with patients' responses were shown, but without any score values shown for the 12 response alternatives. Participants were asked to rank the responses according to the severity of symptoms, from the response with the highest to the lowest negative impact on QoL. The ranking was then transformed to the corresponding score values in the original score (CIS).

Based on the results of a previous similar study regarding the pouch dysfunction score^10^, it was hypothesized that the selection of the participants to the correct items above the threshold of random selection would not be high, and that of the five professional groups stoma nurses would have the best knowledge and estimation of the stoma‐related problems because they spent most time with patients with a stoma.

### Statistical analysis

All statistical analyses were performed using STATA® software version 12 (StataCorp, College Station, Texas, USA) and Excel® software (Microsoft, Redmond, Washington, USA). Descriptive data were presented as numbers and percentages. The χ^2^ test was used to compare performance in choosing the items corresponding to those in the original CIS between different professional groups. The Kruskal–Wallis H test was used to compare the response values between the different professional categories. The responses of all other professionals were compared with those of stoma nurses; the hypothesis was that stoma nurses would have better knowledge than the other groups.

Agreement between healthcare personnel and patients was classified as a good, moderate or poor match. A good match was achieved when the response option was rated with an identical value or different by no more than 1 point from that of the original CIS. A moderate match was attained when the value differed by 2 points, and a poor match was designated if the value differed by more than 2 points. The threshold of random selection was defined as each item would have the same probability of being chosen (7 of 17 = 0·41), which equates to a frequency of 64 of 157 random selections (number of participants). *P* < 0·050 was considered statistically significant.

## Results

**Figure 1:**
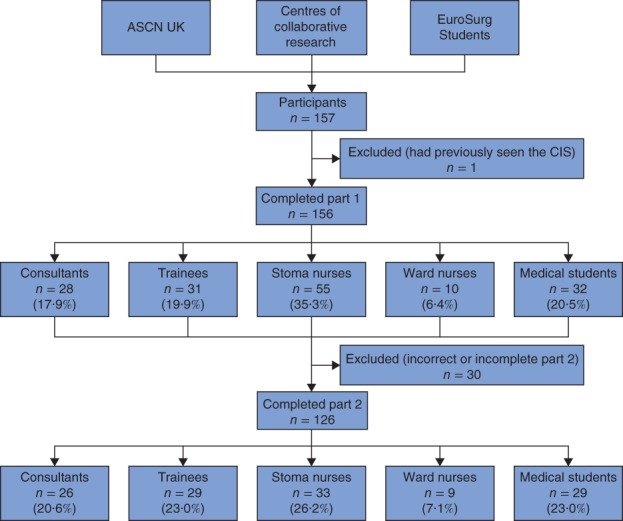
Flow chart of the participation process. ASCN, Association of Stoma Care Nurses; CIS, Colostomy Impact Score

**Figure 2:**
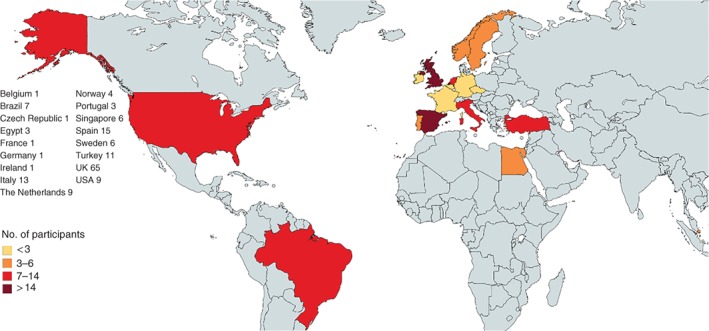
Participating countries and numbers of participants

A total of 157 healthcare professionals returned the surveys. Data from one respondent were excluded as this person had previous knowledge of the CIS score. The flow chart of the participation process is shown in *Fig*. 1. Some 84·0 per cent of participants were European and 16·0 per cent were from the Americas, Asia and Africa (*Fig*. 2). The distribution of the 157 professionals between countries and their experience are shown in *Table* 1. There was clear predominance of stoma nurses from the UK, representing 71 per cent of this professional category.

**Table 1:** Distribution of professional groups among participating countries, with years of experience

	No. of personnel (*n* = 157)	Stoma nurses (*n* = 55)	Consultants (*n* = 29)	Trainees (*n* = 31)	Ward nurses (*n* = 10)	Medical students (*n* = 32)
Country						
Belgium	1	1 (2)	0 (0)	0 (0)	0 (0)	0 (0)
Brazil	7	2 (4)	2 (7)	2 (6)	1 (10)	0 (0)
Czech Republic	1	0 (0)	0 (0)	0 (0)	0 (0)	1 (3)
Egypt	3	0 (0)	2 (7)	1 (3)	0 (0)	0 (0)
France	1	0 (0)	0 (0)	0 (0)	0 (0)	1 (3)
Germany	1	0 (0)	0 (0)	0 (0)	0 (0)	1 (3)
Ireland	1	1 (2)	0 (0)	0 (0)	0 (0)	0 (0)
Italy	13	0 (0)	1 (3)	4 (13)	0 (0)	8 (25)
The Netherlands	9	1 (2)	3 (10)	4 (13)	0 (0)	1 (3)
Norway	4	0 (0)	2 (7)	0 (0)	2 (20)	0 (0)
Portugal	3	1 (2)	2 (7)	0 (0)	0 (0)	0 (0)
Singapore	6	1 (2)	3 (10)	0 (0)	1 (10)	1 (3)
Spain	15	4 (7)	3 (10)	1 (3)	0 (0)	7 (22)
Sweden	6	0 (0)	3 (10)	1 (3)	2 (20)	0 (0)
Turkey	11	2 (4)	2 (7)	2 (6)	2 (20)	3 (9)
UK	65	39 (71)	3 (10)	14 (45)	0 (0)	9 (28)
USA	9	3 (5)	2 (7)	2 (6)	2 (20)	0 (0)
Other	1	0 (0)	1 (3)	0 (0)	0 (0)	0 (0)
Experience (years)*						
< 1		0 (0)	2 (7)	10 (32)	0 (0)	1 (3)
1–3		8 (15)	7 (24)	5 (16)	1 (10)	4 (13)
3–5		7 (13)	2 (7)	10 (32)	0 (0)	17 (53)
> 5		40 (73)	18 (62)	6 (19)	9 (90)	10 (31)

Values in parentheses are percentages.

* Based on the question: ‘How many years have you been in your current profession?’.

The response rate from the invited international collaborators and the EuroSurg group was 50·4 per cent (116 responses from 230 invitations). The total number of individual e‐mail invitations sent to the ASCN UK was not known.

### Step 1: rating of colostomy‐related problems

**Figure 3:**
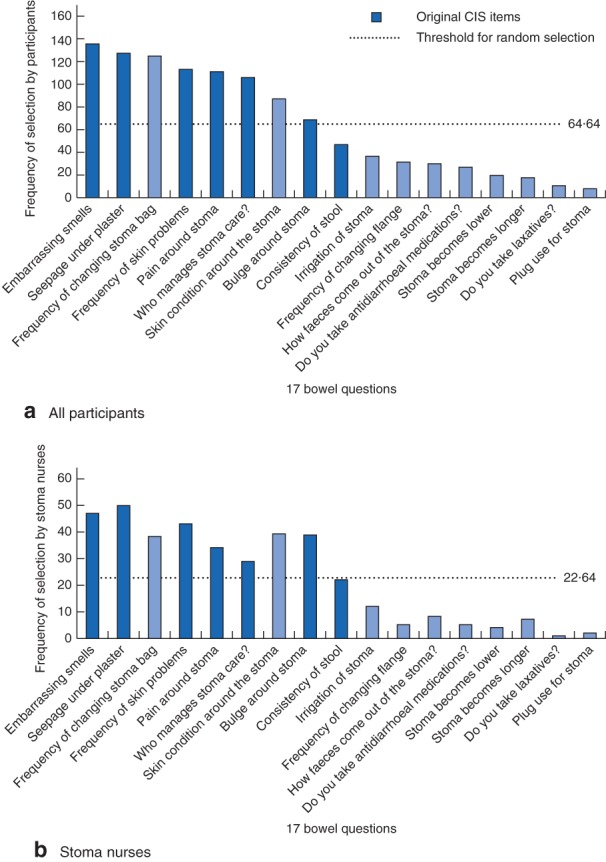
Frequency of selection of the 17 items presented to patients for the development of the Colostomy Impact Score (CIS) by **a** all participants and **b** stoma nurses. No significant difference was found between all participants and stoma nurses

The 17 items of the colostomy‐related problems and their frequency of selection by the participants are shown in *Fig*. 3. Six of the original seven items of the CIS showed frequencies above the threshold for random selection, and five items were selected in the top seven frequencies of selection (*Fig*. 3 *a*). These items included embarrassing smells, seepage under sticking plaster, frequency of skin problems, pain around stoma and managing stoma care. However, two items included in the CIS, stool consistency and parastomal bulging, were underestimated by the participants, and two items not included in the CIS, frequency of changing stoma bag and condition of skin around the stoma, were among the seven most reported items and were clearly higher than the threshold for random selection. The overall percentage of participants who had chosen five or more of the original items in the CIS was 57·1 per cent. No statistically significant difference was observed between the five professional groups (*P* = 0·079). When comparing every other professional category with stoma nurses, the only group that performed significantly worse were medical students, *P* = 0·010 (*Table* 2).

**Table 2:** Correct selections for stoma nurses versus other healthcare personnel

	Stoma nurses (*n* = 55)	Consultants (*n* = 28)	Trainees (*n* = 31)	Ward nurses (*n* = 10)	Medical students (*n* = 32)	Total (*n* = 156)
No. of correct selections						
≤ 4	19 (35)	13 (46)	10 (32)	5 (50)	20 (63)	67 (42·9)
≥ 5	36 (65)	15 (54)	21 (68)	5 (50)	12 (38)	89 (57·1)
*P*		0·293*	0·829*	0·352*	0·010*	
		0·118†				

Values in parentheses are percentages.

* Stoma nurses *versus* each category;

† stoma nurses *versus* the other four categories combined (χ^2^ test).

### Step 2: perception of the impact of CIS items on quality of life

**Figure 4:**
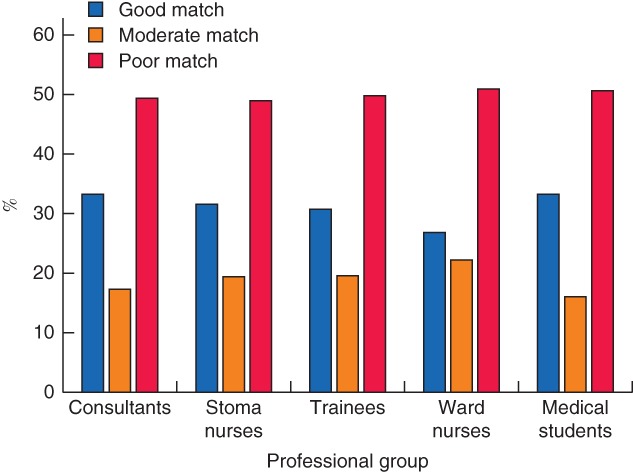
Response matching to original Colostomy Impact Score (CIS) values by professional group. A similar pattern was seen for matching when responses for all professional groups combined were compared with the original CIS values

Some 80·8 per cent of participants (126 of 156) completed the second part of the survey. The percentages of good, moderate and poor matches for the values assigned to CIS responses by each professional group are shown in *Fig*. 4. The answers of 49·7 per cent of participants matched poorly the original score values. Only 31·8 per cent of participants showed a good match with the values of the original score.

**Figure 5:**
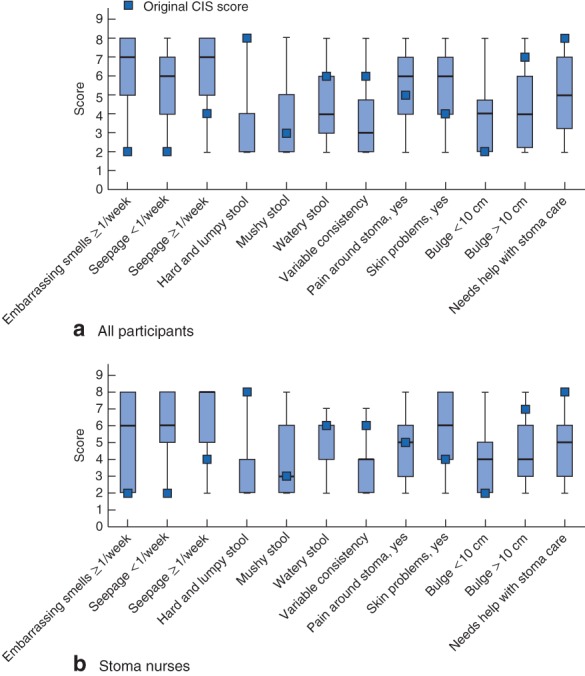
Score assigned by **a** all participants and **b** stoma nurses for each of the 12 responses to the seven factors in the Colostomy Impact Score (CIS) compared with original scores. Median values, interquartile ranges and ranges are denoted by bars, boxes and error bars respectively for every response value scored in step 2. The pattern was similar for all participants and stoma nurses

Median (i.q.r.) values of the scores assigned by participants to each of the 12 responses compared with the CIS are presented in Fig. 5. There was clear incongruity between the original score and the participants' median estimated values for seven responses, whereas for five responses the original score value lay within the interquartile range of the participants' estimate.

Hard and lumpy stool consistency was the most underestimated response. Embarrassing smells once a week or more frequently was the most overestimated.

No significant difference was observed when the different professional groups were compared with one another (*P* = 0·108).

The correlation between country or experience and matching was not investigated owing to the relatively small sample size. Some countries and categories had only one participant.

## Discussion

This study has shown significant discrepancy in the perception of stoma‐related problems between healthcare professionals and patients with a stoma. Almost half of replies of the participating healthcare professionals had a poor match with the original patient‐derived score.

Stool consistency, particularly hard and lumpy stool, was the item that demonstrated the most incongruity between patients' and participants' perspectives, followed by bulging around the stoma. Others^11^ ^12^ have reported the impact of hard stool consistency on patients' QoL, concluding that, although constipation with increased stool consistency was an often neglected problem among stoma patients, it was considered a strong factor in reducing QoL and increasing the number of stoma clinic visits due to pain. As this problem is often raised at the primary care level and handled with dietary recommendations and/or simple laxatives or aperients, this may explain under‐reporting in secondary care. Bulging around the stoma was underestimated by all professional groups except for stoma nurses. Prevailing surgical dogma that most parastomal hernias are asymptomatic may have contributed to the underestimation^13^ ^14^. Even bulging is not necessarily indicative of herniation, as it may also be associated with prolapse, thick mesentery, excessive subcutaneous length of the stoma or weak abdominal wall^15^, ^16^, ^17^.

The high frequency in respondents' selection of the stoma bag changing frequency item could be attributed to the financial burden of this item. The CIS was developed in Denmark where stoma care is free of individual charge. This may not be the case in all countries.

Several studies have described various discrepancies between physicians' and patients' points of view for a variety of disorders, including colorectal diseases^10^ ^18^, ^19^, ^20^, ^21^, ^22^. Participants in those studies were physicians only. QoL outcomes derived from the physicians' perspective may be imprecise^23^ ^24^. Regarding QoL, the patients' own rating is best.

The main strength of the study is the relatively large number of participants, the diversity of the countries of practice, and the coverage of different professional groups that might be involved in the management of stoma‐related problems. This international perspective of the survey improves the heterogeneity among healthcare professionals in relation to healthcare organization, culture and climate difference.

The study has several limitations. As the recruitment process was confined to centres involved in collaborative research work with Aarhus, one stoma nurse association with predominance of stoma nurses from the UK and one student network, selection bias must be present. As only minor differences were observed between the different professional groups, it is unlikely that a larger study would yield significantly different findings. The number of ward nurses was small, and inferences should be made with caution.

This study should raise awareness and improve the understanding of colostomy problems among healthcare professionals, and prompt closer communication with the patient throughout the treatment and follow‐up process. As the patients' perspectives of the impact of different symptoms on QoL often differ from those of healthcare professionals, patient‐reported outcome measures should be developed to incorporate the patients' view, as was done with the CIS. Use of the CIS could raise awareness of how stomas affect patients' QoL. This is necessary to improve clinical practice and achieve better outcomes for these patients.

## Collaborators

The following are members of the Colostomy Impact Score study group: A. D'Hoore (Belgium), E. Espin (Spain), K. Buzatti (Brazil), M. Wong (Singapore), N. Smart (UK), N. Figueiredo (Portugal), P. Nilsson (Sweden), R. Madoff (USA), S. Wexner (USA), T. Cecil (UK), T. Øresland (Norway), W. Omar (Egypt), W. Borstlap (The Netherlands).

## Supporting information


**Appendix S1** Colostomy Impact Score
**Appendix S2** The 17 questions on stoma‐related problems
**Appendix S3** Participants

## References

[1] American Cancer Society . *Cancer Facts & Figures 2016* https://www.cancer.org/research/cancer-facts-statistics/all-cancer-facts-figures/cancer-facts-figures-2016.html [accessed 8 November 2017].

[2] Feddern ML , Emmertsen KJ , Laurberg S . Life with a stoma after curative resection for rectal cancer: a population‐based cross‐sectional study. Colorectal Dis 2015; 17: 1011–1017. 26112651 10.1111/codi.13041

[3] Lindgren R , Hallböök O , Rutegård J , Sjödahl R , Matthiessen P . What is the risk for a permanent stoma after low anterior resection of the rectum for cancer? A six‐year follow‐up of a multicenter trial. Dis Colon Rectum 2011; 54: 41–47. 21160312 10.1007/DCR.0b013e3181fd2948

[4] Shabbir J , Britton DC . Stoma complications: a literature overview. Colorectal Dis 2010; 12: 958–964. 19604288 10.1111/j.1463-1318.2009.02006.x

[5] Mahjoubi B , Mirzaei R , Azizi R , Jafarinia M , Zahedi‐Shoolami L . A cross‐sectional survey of quality of life in colostomates: a report from Iran. Health Qual Life Outcomes 2012; 10: 136. 23170951 10.1186/1477-7525-10-136 PMC3511248

[6] Bloemen JG , Visschers RG , Truin W , Beets GL , Konsten JL . Long‐term quality of life in patients with rectal cancer: association with severe postoperative complications and presence of a stoma. Dis Colon Rectum 2009; 52: 1251–1258. 19571701 10.1007/DCR.0b013e3181a74322

[7] Guren MG , Eriksen MT , Wiig JN , Carlsen E , Nesbakken A , Sigurdsson HK *et al*; Norwegian Rectal Cancer Group. Quality of life and functional outcome following anterior or abdominoperineal resection for rectal cancer. Eur J Surg Oncol 2005; 31: 735–742. 16180267 10.1016/j.ejso.2005.05.004

[8] Thyø A , Emmertsen KJ , Pinkney TD , Christensen P , Laurberg S . The colostomy impact score: development and validation of a patient reported outcome measure for rectal cancer patients with a permanent colostomy. A population‐based study. Colorectal Dis 2017; 19: O25–O33. 27883253 10.1111/codi.13566

[9] Harris PA , Taylor R , Thielke R , Payne J , Gonzalez N , Conde JG . Research electronic data capture (REDCap) ‐ a metadata‐driven methodology and workflow process for providing translational research informatics support. J Biomed Inform 2009; 42: 377–381. 18929686 10.1016/j.jbi.2008.08.010 PMC2700030

[10] Brandsborg S , Chen TY , Nicholls RJ , Laurberg S . Difference between patients' and clinicians' perception of pouch dysfunction and its impact on quality of life following restorative proctocolectomy. Colorectal Dis 2015; 17: O136–O140. 25773269 10.1111/codi.12948

[11] Krokowicz L , Bobkiewicz A , Borejsza‐Wysocki M , Kuczynska B , Lisowska A , Skowronska‐Piekarska U *et al* A prospective, descriptive study to assess the effect of dietary and pharmacological strategies to manage constipation in patients with a stoma. Ostomy Wound Manage 2015; 61: 14–22. 27763879

[12] Krokowicz Ł , Sławek S , Ledwosiński W , Bobkiewicz A , Borejsza‐Wysocki M , Kuczyńska B *et al* Surgical methods of treatment of intestinal passage disturbances with the characteristics of constipation in patients with intestinal stoma based on own experience. Pol Przegl Chir 2015; 87: 160–165. 26146114 10.1515/pjs-2015-0038

[13] Pearl RK . Parastomal hernias. World J Surg 1989; 13: 569–572. 2815801 10.1007/BF01658872

[14] Cross AJ , Buchwald PL , Frizelle FA , Eglinton TW . Meta‐analysis of prophylactic mesh to prevent parastomal hernia. Br J Surg 2017; 104: 179–186. 28004850 10.1002/bjs.10402

[15] Marinez AC , González E , Holm K , Bock D , Prytz M , Haglind E *et al* Stoma‐related symptoms in patients operated for rectal cancer with abdominoperineal excision. Int J Colorectal Dis 2016; 31: 635–641. 26728024 10.1007/s00384-015-2491-4

[16] McGee MF , Cataldo PA . Intestinal stomas In The ASCRS Textbook of Colon and Rectal Surgery [Internet], Steele SR, Hull TL, Read TE, Saclarides TJ, Senagore AJ, Whitlow CB (eds). Springer International Publishing: Cham, 2016; 971–1013.

[17] Kald A , Juul KN , Hjortsvang H , Sjödahl RI . Quality of life is impaired in patients with peristomal bulging of a sigmoid colostomy. Scand J Gastroenterol 2008; 43: 627–633. 18415759 10.1080/00365520701858470

[18] Miravitlles M , Ferrer J , Baró E , Lleonart M , Galera J . Differences between physician and patient in the perception of symptoms and their severity in COPD. Respir Med 2013; 107: 1977–1985. 23890959 10.1016/j.rmed.2013.06.019

[19] Platt FW , Keating KN . Differences in physician and patient perceptions of uncomplicated UTI symptom severity: understanding the communication gap. Int J Clin Pract 2007; 61: 303–308. 17263717 10.1111/j.1742-1241.2006.01277.x

[20] Rodríguez LV , Blander DS , Dorey F , Raz S , Zimmern P . Discrepancy in patient and physician perception of patient's quality of life related to urinary symptoms. Urology 2003; 62: 49–53. 12837421 10.1016/s0090-4295(03)00144-4

[21] Yen JC . Patient–Physician Discordance in Systemic Lupus Erythematosus and its Impact on Medication Adherence and Alternative Medicine Use [Internet] *. ProQuest Dissertations and Theses*; 2002 http://search.proquest.com.ezp-prod1.hul.harvard.edu/docview/305461354?accountid=11311%5Cnhttp://sfx.hul.harvard.edu/sfx_local?url_ver=Z39.88-2004&rft_val_fmt=info:ofi/fmt:kev:mtx:dissertation&genre=dissertations+&+theses&sid=ProQ:ProQuest+Dissertations+& [accessed 27 March 2018].

[22] Chen TY , Emmertsen KJ , Laurberg S . Bowel dysfunction after rectal cancer treatment: a study comparing the specialist's *versus* patient's perspective. BMJ Open 2014; 4: e003374. 10.1136/bmjopen-2013-003374 PMC3902194 24448844

[23] Undén AL , Elofsson S . Health from the patient's point of view. How does it relate to the physician's judgement? Fam Pract 2001; 18: 174–180. 11264268 10.1093/fampra/18.2.174

[24] Woodend AK , Nair RC , Tang AS . Definition of life quality from a patient *versus* health care professional perspective. Int J Rehabil Res 1997; 20: 71–80. 9089016 10.1097/00004356-199703000-00006

