# Supplementary material for: Patient and healthcare professional perceptions of colostomy‐related problems and their impact on quality of life following rectal cancer surgery
Source: BJS Open. 2018 May 7;2(5):336–44. doi: 10.1002/bjs5.69 (PMC6156164; doi:10.1002/bjs5.69)
Supplement: Supplementary file 1 — Appendix S1 Colostomy Impact Score Appendix S2 The 17 questions on stoma‐related problems Appendix S3 Participants [file BJS5-2-336-s001.docx]

**BJS5_69**

**Patient and healthcare professional perceptions of colostomy-related problems and their impact on quality of life following rectal cancer surgery**

**H. Elfeki, A. Thyø, D. Nepogodiev, T. D. Pinkney, M. White, S. Laurberg and P. Christensen**

**Appendix S1** Colostomy Impact Score

**The Colostomy Impact Score: Scoring instructions.**

Please add the scores from each 7 answers to one final score.

**Do you experience embarrassing smells from your stoma-bag?**

☐ No, never 0

☐ Yes, less than once a week 0

☐ Yes, at least once a week 2

**Do you experience seepage of faeces under the sticking plaster?**

☐ No, never 0

☐ Yes, less than once a week 2

☐ Yes, at least once a week 4

**What is the consistency of your faeces?**

☐ Hard and lumpy 8

☐ formed and soft 0

☐ Mushy 3

☐ Watery 6

☐ Variable 6

**Do you ever experience pain in and around the stoma?**

☐ No, never 0

☐ Yes 5

**Do you experience any skin problems around your stoma?**

☐ No, never 0

☐ Yes 4

**Have you noticed a bulge around the stoma?**

☐ No 0

☐ Yes, I have a small bulge (under 10 cm) 2

☐ Yes, I have a larger bulge (over 10 cm) 7

**Who manages your stoma care?**

☐ I do it all myself 0

☐ I need support and instruction 8

**Total score** -----------

**Interpretation:**

***0-9***: Minor Colostomy Impact

***10-38***: Major Colostomy Impact

**Appendix S2** The 17 questions on stoma-related problems

**Questions**

**Q1.Do you experience embarrassing smells from your stoma-bag?**

- No, never
- Yes, less than once a month
- Yes, 1-4 times a month
- Yes, 1-6 times a week
- Yes, daily

**Q2.Do you experience seepage of feces under the sticking plaster?**

- No, never
- Yes, less than once a month
- Yes, 1-4 times a month
- Yes, 1-6 times a week
- Yes, daily

**Q3.Do you take laxatives?**

- No
- Yes

**Q4.Do you take anti-diarrheal medication?**

- No
- Yes

**Q5.How often do you have to change the stoma-bag?**

- More than 4 times per day
- 2-4 times per day
- 1 time per day
- 1-6 times per week
- Less than once a week

**Q6.If you use a two-part bandage, how often do you change the stoma flange?**

- More than once a day
- Once a day
- 4-6 times per week
- 1-3 times per week
- Less than once a week
- Don’t use 2-part bandages.

**Q7.Have you noticed how your feces come out of the stoma?**

- No, I haven’t noticed how the feces come out of the stoma.
- Yes, it comes out like a normal motion.
- Yes, it comes out like a pencil thin “sausage”.
- Yes, it comes out in a seeping fashion over a long period of time.
- Yes, it comes out explosively.

**Q8.What is the consistency of your feces?**

- Hard and lumpy
- Soft and formed
- Mushy
- Watery
- Variable

**Q9.Have you noticed if your stoma has become lower?**

- No.
- Yes, it has sunken in a little.
- Yes, it has sunken in somewhat.
- Yes, it has sunken in a lot.
- Yes it has sunken in completely.

**Q10.Have you noticed if your stoma has become longer?**

- No
- Yes, a little
- Yes, somewhat
- Yes, a lot

**Q11.Do you experience pain in and around the stoma?**

- No, never
- Yes, less than once a month
- Yes, 1-4 times a month
- Yes, 1-6 times a week
- Yes, daily

**Q12.How is the skin around your stoma?**

- Normal, like the skin on the rest of my body
- Reddened.
- Itchy and reddened
- Chafed
- With bleeding sores

**Q13.Do you experience any skin problems around your stoma?**

- No, never
- Yes, rarely
- Yes, from time to time
- Yes, often
- Yes, all the time

**Q14.Have you noticed a bulge around the stoma?**

- No
- Yes, I have a small bulge (under 10cm)
- Yes, I have a larger bulge (over 10cm)

**Q15.Who manages your stoma care?**

- I do it all myself
- I need support and instruction
- I can change the bag myself, but I need help changing the flange
- I am receiving training in stoma care from the district nurses.
- I get help with all my stoma care

**Q16.Do you use a plug in your stoma?**

- No, never
- Yes, occasionally
- Yes, a few hours every day
- Yes, most of the time
- Have tried it, it doesn’t work

**Q17.Do you use lavage treatment (irrigation) of the large intestine through your stoma?**

- No, I have never had it offered
- No, I don’t want it
- I have tried it, but it has stopped
- Yes, I use it regularly

**Appendix S3** Participants

Aditya Borakati

Albert Wolthuis

Alejandro Solís Peña

Alexios Tzivanakis

Amanda Gunning

Amirul Ikhwan Adlan

Anais Sánchez

Andrea Simioni

Andrew Bird

Aneel Bhangu

Anita Van den Bosch

Anna Moseley

Anne Gottwalt

Anne Sophie van Dalen

Araceli Varo Muñoz

Ariella Altman

Arthur Manoel Braga

Audrey Steele

Ayla

Aziz Ihsan Tavuz

Bahar Busra Ozkan

Barbara Langenhoff

Basak Yuksek

Beatriz Deoti e Silva

Rodrigues

Benjamin France

Bruno Sensi

Carol Loi

Caroline Nordenvall

Catherine Brooks

Cheryl Wells

Chetan Khatri

Chris Robinson

Cihangir Akyol

Claire Bohr

Colette

Conor S Jones

Cristina Navalon Perez

Cristina Perez Costoya

David Golding

De Groof

Debbie Li

Di Kay

Durucan Aydin

Ed Fitzgerald

Elaine Swan

Eloy Espin

Emma Maltby

Esra Çelebi

Ethem Gecim

Evelina Woin

Federica Elena Cazzola

Francesco Pata

Francielle Rodrigues

Franco Marinello

G. J. Veldink

Gabriel Garcia

Gaetano Gallo

Gail

Gail Booth

Gerardo Perrotta

Gianluca Pellino

Gill Skipper

Glicerio Moura-Tebbe

Gloria Vivas Edo

Grace Pike

Heidi Miltvedt

Hugo Domingos

Ian Daniels

Immaculada Davin

Iskandar Rakhimov

James C Glasbey

Jane Andréa Vieira Novaes

Jane Thacker

Jarno Melenhorst

Jason

Jay Bradbury

Jen

Jeneva Ebba

Jennie Burch

Jenny Marsden

Joan Garwood

Johan Erlandsson

Johannes Kurt Schultz

Josh Burke

Juan Viñas-Salas

Judy Hanley

Julián Serrano Carmona

Katarina Friström

Kelly Cristine de Lacerda Rodrigues Buzatti

Kenneth Keogh

Kirstie Kaye

Laura Gavagna

Linda Crutch

Louise Rafferty

Luca Turati

Lynn Huemann

Lynne Macinnes

Maddie White

Marcela Monteiro Pinheiro

Mark Wong

Marta de la Rosa Estadella

Mary Grace Francisco

Mary Kwaan

Mary W Quigley

Matteo Bevilacqua

Mel

Melanie Jerome

Merle Stellingwerf

Michael Saat

Michelle Boucher

Mike Bath

Monica Lilja

Monika Egenvall

Mostafa Shalaby

Nadia Abuhussein

Nazir Naimy

Neil Smart

Ng Jia Lin

Nuno Figueiredo

Nurtaç Özkayalarlı

Olga Anabitarte Bautista

Ong Choo Eng

Pamela Rahmann

Pavol Vasko

Per J Nilsson

Pia-Elena Frey

Pietro Maria Naccari

Pip Chandler

Priyesh Chauhan

Raquel Rodríguez García

Rémy Chevalier

Richard Wilkin

Robert Madoff

Rosanna van Langen

Rutger Stijns

Ruth Blanco Colino

Sameh H Emile

Samuel

Simon Turley

Sivesh K Kamarajah

Stephen Chapman

Steve de Castro

Suleyman Utku Celik

Susan Field

Suzie Dukes

Tatiana Vasconcelos Quaresma

Therese Andersson

Thomas Drake

Tom Cecil

Valeria

Vicky Burrows

Vicky Preece

Virginia Hill

Waleed Omar

Yasemin Köşker

Yvette Perston
